# Analysis of codon usage pattern in *Taenia saginata* based on a transcriptome dataset

**DOI:** 10.1186/s13071-014-0527-1

**Published:** 2014-12-02

**Authors:** Xing Yang, Xuenong Luo, Xuepeng Cai

**Affiliations:** State Key Laboratory of Veterinary Etiological Biology, Lanzhou Veterinary Research Institute, Chinese Academy of Agricultural Sciences, Lanzhou, 730046 PR China; College of Veterinary Medicine, Jilin University, Changchun, 130000 PR China

**Keywords:** *Taenia saginata*, Codon usage bias, Trancriptome, Optimal codon

## Abstract

**Background:**

Codon usage bias is an important evolutionary feature in a genome and has been widely documented in many genomes. Analysis of codon usage bias has significance for mRNA translation, design of transgenes, new gene discovery, and studies of molecular biology and evolution, etc. However, the information about synonymous codon usage pattern of *T. saginata* genome remains unclear. *T. saginata* is a food-borne zoonotic cestode which infects approximataely 50 million humans worldwide, and causes significant health problems to the host and considerable socio-economic losses as a consequence. In this study, synonymous codon usage in *T. saginata* were examined.

**Methods:**

Total RNA was isolated from *T. saginata* cysticerci and 91,487 unigenes were generated using Illumina sequencing technology. After filtering, the final sequence collection containing 11,399 CDSs was used for our analysis.

**Results:**

Neutrality analysis showed that the *T. saginata* had a wide GC3 distribution and a significant correlation was observed between GC12 and GC3. NC-plot showed most of genes on or close to the expected curve, but only a few points with low-ENC values were below it, suggesting that mutational bias plays a major role in shaping codon usage. The Parity Rule 2 plot (PR2) analysis showed that GC and AT were not used proportionally. We also identified twenty-three optimal codons in the *T. saginata* genome, all of which were ended with a G or C residue. These results suggest that mutational and selection forces are probably driving factors of codon usage bias in *T. saginata* genome. Meanwhile, other factors such as protein length, gene expression, GC content of genes, the hydropathicity of each protein also influence codon usage.

**Conclusions:**

Here, we systematically analyzed the codon usage pattern and identified factors shaping in codon usage bias in *T. saginata.* Currently, no complete nuclear genome is available for codon usage analysis at the genome level in *T. saginata*. This is the first report to investigate codon biology in *T. sagninata*. Such information does not only bring about a new perspective for understanding the mechanisms of biased usage of synonymous codons but also provide useful clues for molecular genetic engineering and evolutionary studies.

## Background

Codon usage bias (CUB) refers to the phenomenon where synonymous codons are not used with equal frequencies during translation of genes. CUB is a common phenomenon in a wide variety of organisms, including prokaryotes and eukaryotes [1–3]. Many factors have been reported to influence codon usage in various organisms. Weak natural selection and mutational pressure are thought to be the main factors that account for the codon usage variation among the genes in these organisms [4]. Genome-wide investigations of codon usage patterns has an immense importance in understanding the basic features of molecular organization of a genome. In addition, analysis of CUB has many other important applied aspects, such as heterologous gene expression [5], the determining of the origins of species [6], the design of degenerate primers [7], the prediction of expression level of genes [8,9], as well as the prediction of gene functions [10]. However, most of numerous reports on CUB have focused on model organisms and many microorganisms, such as *Caenorhabditis, Drosophila, Arabidopsis* [11]*, yeast* [12]*, Giardia lamblia* [13]*, Entamoeba histolytica* [14]*, Streptomyces* [15]*, Borrelia burgdorferi* [16]*, and Saccharomyces cerevisiae* [17]. For example, in *C. elegans* it is observed that most favored codons are ended with G and/or C (majority are C ending) [18]. In contrast, there are few studies on tapeworms. *T. saginata* is an important parasitic tapeworm which is widely distributed in the world [19]. The adult worms mainly parasitize in the small intestines of humans [20,21]. *T. saginata* can cause great economic losses and endangers public health [22,23]. However, the information about synonymous codon usage pattern of *T. saginata* remains unclear. In this study, we investigated the codon usage profile of *T. saginata* through transcriptome data using a multivariate statistical analysis. Analysis of codon usage pattern in *T. saginata* would provide a basis for understanding the related mechanism for biased usage of synonymous codons and for choosing appropriate host expression systems for an optimized expression of target genes.

## Methods

### Ethics statement

This study was approved by the Animal Ethics Committee of Lanzhou Veterinary Research Institute, Chinese Academy of Agricultural Sciences (Approval No. LVRIAEC2009-2012). The cattle from which *Taenia saginata* cysticerci were collected for transcriptome sequencing, were handled in accordance with good animal practices required by the Animal Ethics Procedures and Guidelines of the People's Republic of China.

### RNA extraction, cDNA library preparation and Illumina sequencing

Total RNA was extracted from cysticerci using the Trizol reagent (Invitrogen, Carlsbad,CA), following the manufacturer^’^s instructions. The quantity and quality of total RNA was analyzed using Agilent 2100 RNA Nanochip (Agilent, Santa Clara, CA, USA) and gel electrophoresis. A total of 16.1 μg of RNA was pooled for the preparation of the cDNA library.

The OligoTex mRNA mini kit (Qiagen) was used to poly-T+ RNA after total RNA was collected according to the manufacturer^’^s protocol. The mRNA was mixed with fragmentation buffer and fragmented into short fragments. cDNA was synthesized using the mRNA fragments as templates. Short fragment (200 ± 25 bp) were gel extracted from an agarose gel and PCR amplified for 15 cycles. Finally, the library was sequenced using the Illumina HiSeq 2000 sequencer (Beijing Genomics Institute, BGI, Shenzhen, Guangdong, China).

### *De novo* assembly

Using Solexa/Illumina RNA-seq deep sequencing technology, we obtained a total of 55.49 million raw reads (4.99 Gb). Further, raw reads were filtered to remove the low-quality reads. The filtration steps were as follows: 1) remove adaptor sequence; 2) remove reads containing the unknown nucleotide “N” over 10%; 3) remove low quality reads containing more than 10 bases with Q-value ≤ 20. Then, the remaining high-quality reads were used for further analysis. Transcriptome raw reads dataset has been submitted to the NCBI (http://www.ncbi.nlm.nih.gov/bioproject/PRJNA260140).

### Sequence data

In this study, a total of 91,487 *T. saginata* unigenes were obtained. Based on a sequence similarity with known proteins, a total of 59,262 unigenes were annotated. Up to 57,607 of which were annotated against the NCBI non-redundant (Nr) protein database, 24,860 were assigned to the protein database Clusters of Orthologous Groups (COG), 26,476 were assigned to the term annotation database of Gene Ontology (GO), and 43,575 were assigned to 200 pathways in the database of Kyoto Encyclopedia of Genes and Genomes (KEGG). Among the annotated unigenes, 61,941 coding sequences (CDS) were obtained by the BLASTx algorithm [24]. All CDSs were analyzed using the FrameDP software [25], which has the ability to self-train directly on EST clusters instead of requiring curated cDNA sets to train the underlying ESTScan and DECODER software [26].

To minimise the sampling error, only CDS sequences longer than 300 bp were used for this study. The final sequence collection containing 11,399 CDSs was used for our analyses.

### Indices of codon usage

Codon usage in these genes was assessed using the program codonW 1.4.4 (J Peden, http://codonw.sourceforge.net). Relative synonymous codon usage (RSCU) is the observed frequency of a codon divided by the frequency expected, if all synonyms for that amino acid were used equally [27]. Thus, RSCU values close to 1.0 indicate lack of bias whereas values more than 1 indicates that a codon was used more frequently than expected, while the converse is true for RSCU values less than 1. The effective number of codons (ENC) method was used to quantify the absolute codon usage bias of a CDS [28]. The values of ENC range from 20 (when only one codon is used per amino acid) to 61 (when all codons are used in equal probability). The predicted values of ENC were calculated as

$$ ENC=2+s+\frac{29}{s^2+\left(1-{s}^2\right)} $$

where s represents the given (G + C)_3_ % value [28].

To determine the preferred codon for each synonymous codon group, the ‘relative synonymous codon usage’ RSCU values were calculated according to the formula of previous reports [27].

$$ RSCU=\frac{gij}{{\displaystyle \sum_j^{ni}gij}}nj $$

where g*ij* is the observed number of the *i*th codon for *j*th amino acid which has *n*_*i*_ type of synonymous codons. The codon with RSCU value more than 1.0 has positive codon usage bias, while the value <1.0 has relative negative codon usage bias. When RSCU value is equal to 1.0, it means that this codon is chosen equally and randomly.

The GC content of first, second and third codon position (GC1, GC2 and GC3 respectively) were then calculated. GC12 is the average of GC 1 and GC2, and was used for analysis of neutrality plots (GC12vsGC3) [29]. The codon adaptation index (CAI) was used to estimate the extent of bias toward codons that were known to be preferred in highly expressed genes. A CAI value is between 0 and 1.0, and a higher value means a likely stronger codon usage bias and a potential higher expression level [30].

### Correspondence analysis(CA)

Correspondence analysis (CA) has been widely used to explore codon usage variation among genes. CA is a sophisticated multivariate statistical technique in which the codon usage data (59 codons) are plotted in a multidimensional space of 59 axes (excluding Met, Trp and stop codons) and then it identifies the axes which represent the most prominent factors contributing to variation among genes [31,32].

### Determination of optimal codons

We selected 5% of the total genes with extremely high and low CAI values which were regarded as the high and low expression genes datasets, respectively. Codon usage was compared using Chi squared contingency test of the two groups, and codons whose frequency of usage were significantly higher (P < 0.01) in highly expressed genes than in genes with low level of expression would be defined as the optimal codons [33].

### Statistical analysis

CodonW 1.4.4 software was used to analyze the indices of codon usage. Correlation analysis was carried out using the Spearman’s rank correlation analysis method wrapped in the multianalysis software SPSS version 19.0.

## Results

### Codon usage in *T. saginata*

The pattern of synonymous codon usage in the *T. saginata* has been shown in Table 1. The genomic G + C content for *T. saginata* is 43.61%. Although the genome would thus appear to be slightly A + T rich, overall codon usage is biased toward C- and G-ending codons (32 codons were frequently used codons, 18/32 of the frequently used codons end with G or C), suggesting the compositional constraints are not the most important factor in shaping the codon usage variation among the genes.

**Table 1:** **Codon usage in** ***T. saginata and T. pisiformis***

		***T. saginata***		***T. pisiformis***				***T. saginata***		***T. pisiformis***	
**AA**	**Codon**	***N***	**RSCU**	***N***	**RSCU**	**AA**	**Codon**	***N***	**RSCU**	***N***	**RSCU**
Phe	UUU	99671	0.97	33874	0.93	Ser	**UCU**	89398	1.11	26049	1.12
	**UUC**	106107	1.03	39075	1.07		**UCC**	97503	1.21	27379	1.18
Leu	UUA	38856	0.45	12571	0.41		**UCA**	82438	1.02	23335	1
	**UUG**	97522	1.13	33937	1.12		UCG	72259	0.9	21063	0.9
	**CUU**	107259	1.24	39632	1.3	Pro	**CCU**	85183	1.1	25765	1.12
	**CUC**	119595	1.38	41428	1.36		**CCC**	79819	1.03	23072	1.01
	CUA	52511	0.61	17904	0.59		**CCA**	88760	1.14	26002	1.13
	**CUG**	103594	1.2	36893	1.21		CCG	56540	0.73	16969	0.74
Ile	**AUU**	109448	1.3	41232	1.32	Thr	**ACU**	88533	1.13	29031	1.17
	**AUC**	96607	1.15	36117	1.16		**ACC**	89404	1.14	27437	1.11
	AUA	47039	0.56	16259	0.52		ACA	77048	0.98	23542	0.95
Met	AUG	113948	1				ACG	58709	0.75	18939	0.77
Val	**GUU**	95565	1.12	35811	1.19	Ala	**GCU**	122134	1.21	45524	1.3
	GUC	85970	1	29881	0.99		**GCC**	111542	1.11	38284	1.09
	GUA	45908	0.54	16165	0.54		GCA	95661	0.95	31761	0.91
	**GUG**	115101	1.34	38575	1.28		GCG	73950	0.73	24715	0.7
Tyr	UAU	55514	0.81	21083	0.81	Cys	UGU	55868	0.99	17870	0.96
	**UAC**	81868	1.19	30723	1.19		**UGC**	57522	1.01	19338	1.04
His	CAU	62791	0.95	21227	0.97	Arg	**CGU**	74482	1.33	26854	1.42
	**CAC**	68855	1.05	22602	1.03		**CGC**	67662	1.2	24579	1.3
Gln	CAA	102281	0.96	32614	0.92		**CGA**	67187	1.2	22254	1.18
	**CAG**	110043	1.04	38283	1.08		CGG	41070	0.73	13883	0.74
Asn	**AAU**	111522	1.07	38069	1.05	Ser	AGU	76215	0.94	21586	0.93
	AAC	97493	0.93	34636	0.95		AGC	66542	0.82	20367	0.87
Lys	AAA	115265	0.92	41472	0.88	Arg	AGA	44857	0.8	13883	0.74
	**AAG**	135856	1.08	52821	1.12		AGG	41815	0.74	13184	0.7
Asp	**GAU**	147181	1.1	54522	1.1	Gly	**GGU**	108511	1.38	36512	1.36
	GAC	120957	0.9	44541	0.9		**GGC**	86502	1.1	30139	1.12
Glu	GAA	152714	0.91	56449	0.93		GGA	76898	0.98	27580	1.03
	**GAG**	181548	1.09	65410	1.07		GGG	41741	0.53	13277	0.49

N: the number of codons; RSCU: Relative synonymous codon usage. The frequently used codons are displayed in bold.

In general, the pattern of codon usage is similar among closely related organisms, but differs significantly among distantly related species, such as *Escherichia coli, Saccharomyces cerevisiae and Drosophila melanogaster* [34]. In this study, patterns of codon usage are compared in *T. saginata* and *T. pisiformis* (Table 1) [35], and we found that there are high similarities between them. With the exception of UCA and GGA, the two species have the same preferred codon for all amino acids.

### Nucleotide content of genes

**Figure 1:**
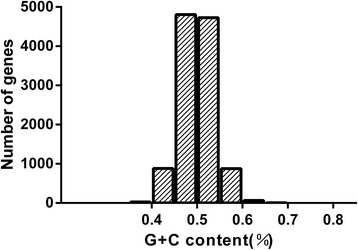
**The distribution of G + C contents in** ***Taenia saginata*** **genes.**

The GC content of the *T. saginata* genes varied from 31% to 80.2% with a standard deviation(SD) of 3.67. The GC content of 11399 genes were mainly distributed between 45% and 55% (Figure 1), this distribution pattern of genes is very similar to *T. pisiformis* [35]. To understand the nucleotide distribution, we concatenated all genes to one sequence, which comprised 532,4389 codons. The GC content in 3 codon positions (GC1, GC2, and GC3) was 0.534, 0.439, and 0.535, respectively. This analysis showed that the GC content at second position is different from the GC content at the first and third position. GC1 was extremely close to GC3, and GC2 was the lowest of all 3 codon positions. The average GC content of all codons was 0.503.

**Figure 2:**
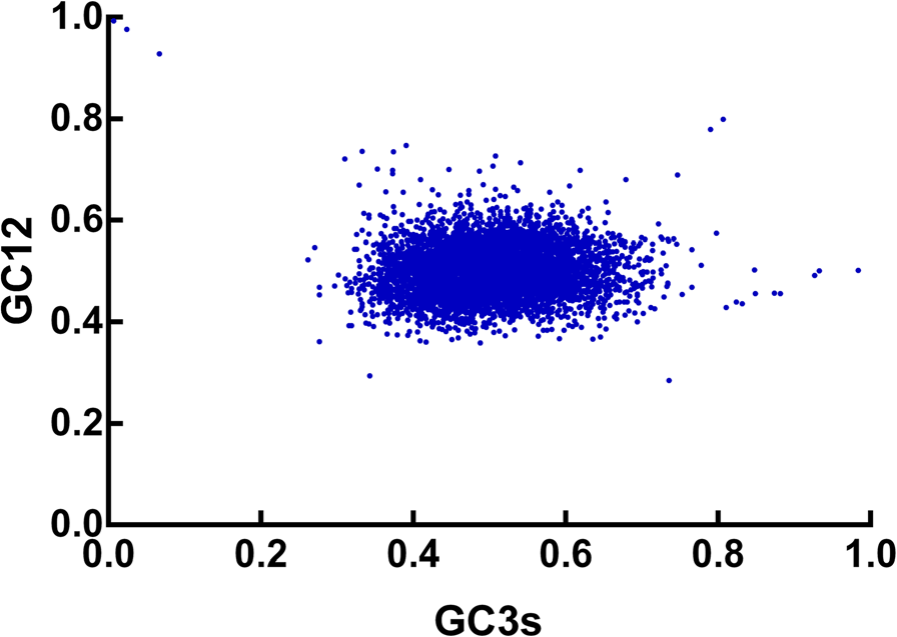
**Neutrality plots (GC12 vs GC3s).**

Neutrality analysis is a useful way to revealing the relationship between GC12 and GC3 and then examining the mutation-selection equilibrium in shaping the CUB. In neutrality plot, if the correlation between GC12 and GC3 is statistically significant and the slope of the regression line is close to 1, mutation bias is assumed to be the main force shaping codon usage. Conversely, selection against mutation bias can cause a narrow distribution of GC content and no correlation between GC12 and GC3 [29,36]. To analyze relations among the three codon positions, neutrality plots (GC12 versus GC3) were performed for *T. saginata*. It was observed that *T. saginata* genes had a wide range of GC3s (0.7 to 98.40) and there is a significant correlation between GC12 and GC3 (r = 0.123, p <0.01) (Figure 2), suggesting mutation and selection are probably both acting to codon usage bias in *T. saginata* genome. In addition, the significantly positive correlation in neutrality plots indicated intragenomic GC mutation bias affects the GC content at all codon positions in a similar manner.

### Relation between ENC and GC3

**Figure 3:**
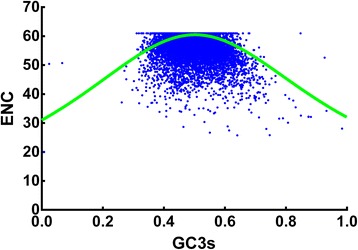
**Distribution of effective number of codons (ENC) and GC3s of** ***Taenia saginata*** **genes.** The solid line (shown in green) indicates the expected ENC value if the codon bias is only due to GC3s.

**Figure 4:**
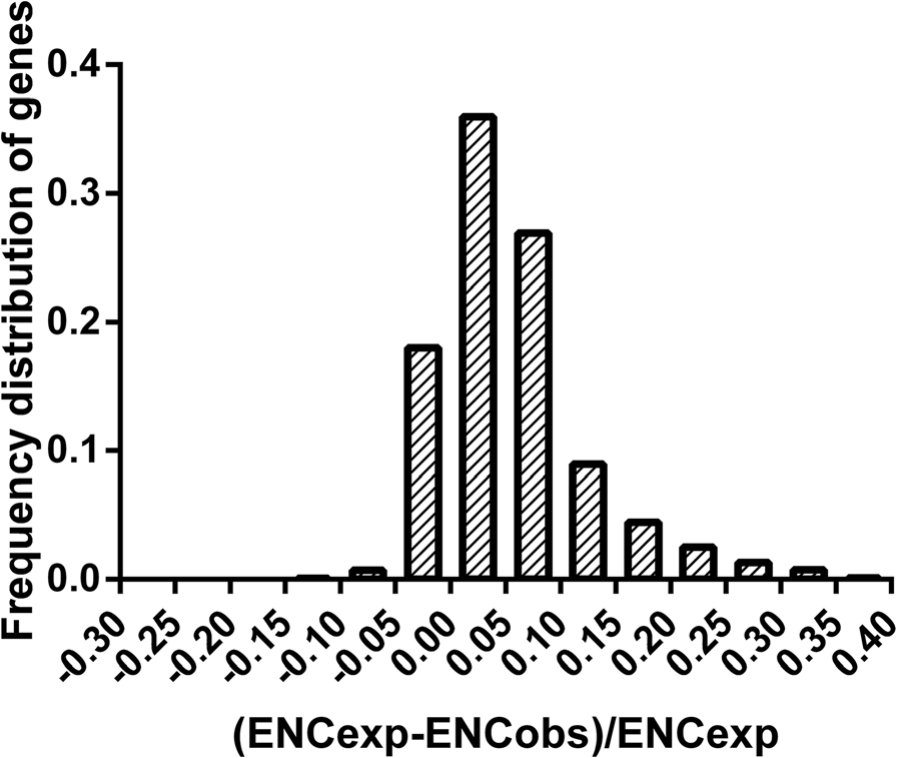
**The frequency distribution of effective number of codons (ENC) ratio.**

To understand the relation between nucleotide composition and codon bias of *T. saginata* sequences, the values of ENC were plotted against the fraction of GC at the third codon position (GC3s) (Figure 3) [28]. The ENC values of different genes ranged from 21.0 to 61, indicating that there are significant differences in codon bias among these genes. From Figure 3 it is obvious that a very considerable proportion of points lies near to the expected curve, which indicates that ENCs of most genes were close to the expected values based on their GC3s. Meanwhile, there are also some points with low ENC lying below the expected curve suggesting these genes have additional codon usage bias that is independent of GC3s. To obtain a more accurate estimate for the difference observed and expected ENC values, we calculated (ENCexp-ENCobs)/ENCexp. The frequency distributions of (ENCexp-ENCobs)/ENCexp are shown in Figure 4. Interestingly, the peak located in 0–0.05 of (ENCexp-ENCobs)/ENCexp value and most genes have −0.05-0.1 of (ENCexp-ENCobs)/ENCexp values indicating that most genes have ENCs slightly difference with expected ENC values from their GC3s. These results suggest that most genes have observed ENCs close to the expected ENC based on GC3s, though a significant number has much lower observed ENCs.

### Correspondence analysis

**Figure 5:**
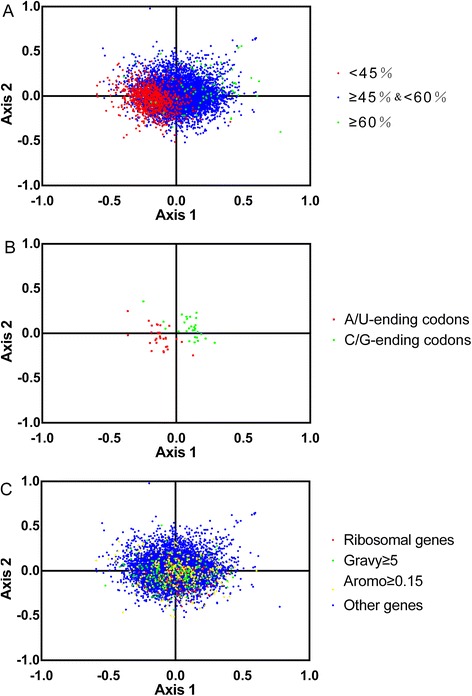
**Correspondence analysis of relative synonymous codon usage for** ***Taenia saginata*** **genes. A**. The distribution of genes is shown along the first and second axes. Green, blue and red dots indicate genes with G + C content more than or equal to 60%, more than or equal to 45%, but less than 60% and less than 45%, respectively. **B**. The distribution of codons on the same two axes as shown in Panel **A**. Codons ending with A and T are shown in red, Codons ending with C and G are shown in green. **C**. Red dots, yellow dots, green triangles and blue dots indicate ribosomal genes, genes with a Aromo value more than or equal to 0.15, genes with a Gravy value higher than 5 and other genes, respectively.

In this thesis, we further investigate the synonymous codon usage variation among genes of *T. saginata* by correspondence analysis in RSCU. The first two axes explain low fractions of the total variation(6.6%and 4.9%,respectively), and the next two axes accounts for 4.1% and 3.3%, respectively. The plot of the first and second axis of each gene is shown in Figure 5A. The distance between genes on the plot is a reflection of their diversity in RSCU. To investigate the effect of GC content of genes on codon usage bias, different GC contents of genes are color coded. The genes, GC content of which is more than or equal to 60%, are plotted in green, and less than 45% is plotted in red. Blue dots indicate the genes which the GC content is between 45 and 60%. In Figure 5A, the high and low GC content of genes separate along the primary axis. Correspondence analysis shows the distribution of genes in the multidimensional space, and also shows the corresponding distribution of synonymous codons (Figure 5B). Figure 5B shows the separation of different bases ending codons along the two axes. The separation of codons on the first axis appears to be largely due to frequency differences in G/C and A/T ending codons. Further calculations revealed a significant correlation (r = 0.6573, P <0.0001) between the GC content of individual genes and their positions on the first axis. In addition, the gene positions on axis 1 were strongly correlated with the GC3s value (r = 0.8253, P <0.0001) and significantly negatively correlated with ENC (r = −0.2322, P <0.0001). From the above results, we found that genes with higher GC and GC3s content values and lower ENC values, which located at the left side of the first axis, indicated a stronger codon bias. This proved that the major factor influencing the codon usage bias among *Taenia saginata* genes was the nucleotide composition of the genes.

In order to analyze the codon usage of different kinds of gene, we selected the hydrophobic genes with gene scores >5, the aromatic genes with gene scores ≥0.15,ribosomal genes and other genes from 11399 genes. The distribution of the four types of genes were shown in Figure 5C. We employed a multivariate analysis of variance (MANOVA) and found that there was a statistically significant difference among four types of genes in codon usage (p < 0.01).

### PR-bias plot

**Figure 6:**
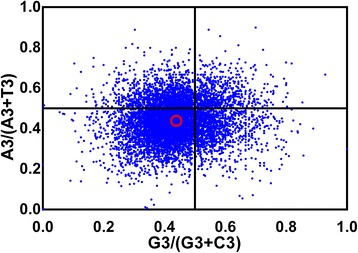
**PR2-bias plot [A3/(A3 + T3) against G3/(G3 + C3)].** Red open circle indicate the average position for each plot. Average position is x = 0.4384 ± 0.09663, y = 0.438562 ± 0.09486.

If mutation bias is the cause of codon bias, GC or AT should be used proportionally among the degenerate codon groups in a gene. In contrast, natural selection for codon choice would not necessarily cause proportional use of G and C (A and T) [37]. To investigate whether these biased codon choices are restricted in highly biased genes, the relation between G and C content and between A and T content in four-fold degenerate codon families were analyzed by PR2 bias plot (Figure 6). The four-codon amino acids are alanine, arginine (CGA, CGT, CGG, CGC), glycine, leucine (CTA, CTT, CTG, CTC), proline, serine (TCA, TCT, TCG, TCC), threonine, and valine. Our results showed that C and T were used more frequently than G and A in *T. saginata*. This observation indicated that both mutation bias and other factors (eg. selection) contribute to codon bias.

### Gene expression level and synonymous codon usage bias

**Figure 7:**
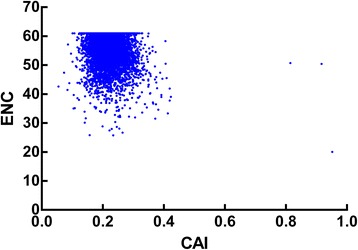
**Plot of ENC versus gene expression level for** ***Taenia saginata*** **.**

Codon adaptation index (CAI) has been extensively used as a predictor of gene expression level [8,9]. The set of reference sequences used for calculating CAI values in this study are genes that encode ribosomal proteins. The expression level of genes of *T. saginata* was assessed through CAI values, which varied from 0.055 to 0.952 with a mean value of 0.22 and a standard deviation of 0.03504. It was found that there was a significantly negative correlation between the gene expression level assessed by CAI value and ENC values (r = −0.1808 and p < 0.0001) (Figure 7), and three significantly positive correlations between CAI value and GC3s, GC content and the positions of genes along axis 1 (r = 0.2437, 0.1009 and 0.4211, respectively, P < 0.0001). The results indicated that the genes with higher expression level had a greater degree of codon usage bias and prefer the codons with C or G at the synonymous position.

### Protein length and synonymous codon usage bias

**Figure 8:**
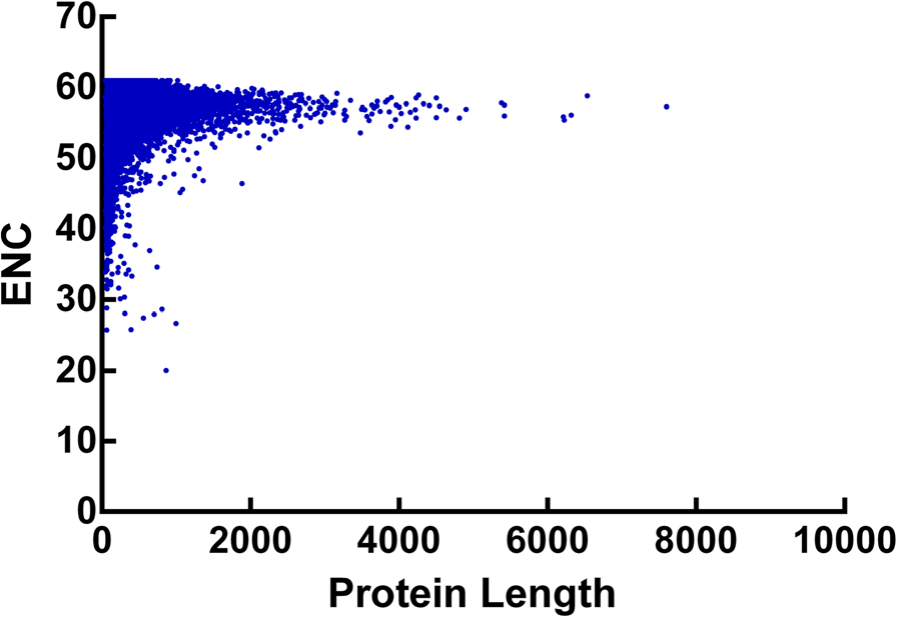
**Plot of ENC versus protein length for** ***Taenia saginata*** **.**

The results of correlation analyses between protein length and axis 1 coordinates, ENC and CAI values showed that the 3 correlation coefficients (*r* = −0.1163, 0.1433 and −0.081, respectively, *P* < 0.01) all significantly correlated (Figure 8), which suggested a general tendency of more biased genes with shorter length to have higher expression level.

### Effect of the hydrophobicity and aromaticity of encoded protein on codon bias

Numerous studies have shown that hydrophobicity and aromaticity of encoded protein play important roles in shaping codon usage of some species. In order to investigate if the same thing is happening to *T. saginata,* we performed a correlation analysis to evaluate whether Gravy and Aromo values were related to ENC values. The correlation analyses between the hydrophobicity of each protein and ENC value showed that the correlation coefficients (r = −0.0883, P <0.001) were significantly correlated. The aromaticity of each protein was not significantly correlated with ENC (r =0.0097, P > 0.05). The analysis results indicated that variation in codon usage were associated with the degree of hydrophobicity, but not with the aromatic amino acids .

### Optimal codons

The average RSCU values of high/low expressed gene sample group are listed in Table 2. Twenty-three codons were determined to be the optimal codons, which were significantly more frequent among the highly expressed genes (P <0.01) according to the chi-square test. Almost all of optimal codons (except GGU and CGU) ended with G or C, indicating that codon usage in *T. saginata* was biased to G- or C-ending synonymous codons.

**Table 2:** **Translational optimal codons of** ***T. saginata***

**AA**	**Codon**	**High RSCU(N)**	**Low RSCU(N)**	**AA**	**Codon**	**High RSCU(N)**	**Low RSCU(N)**
Phe	UUU	0.69 (2341)	1.23 (3032)	Ser	UCU	0.80 (1672)	1.26 (2823)
	UUC*	1.31 (4441)	0.77 (1907)		UCC*	1.51 (3157)	0.87 (1951)
Leu	UUA	0.20 (540)	0.90 (1844)		UCA	0.74 (1540)	1.32 (2967)
	UUG	0.96 (2598)	1.35 (2759)		UCG*	1.06 (2216)	0.79 (1772)
	CUU	0.95 (2570)	1.34 (2750)		AGU	0.85 (1770)	1.03 (2318)
	CUC*	1.97 (5332)	0.79 (1628)		AGC*	1.03 (2150)	0.73 (1640)
	CUA	0.41 (1099)	0.74 (1516)	Pro	CCU	0.91 (1919)	1.21 (2292)
	CUG*	1.52 (4118)	0.88 (1804)		CCC*	1.32 (2776)	0.74 (1399)
Ile	AUU	1.02 (2797)	1.43 (3065)		CCA	0.89 (1875)	1.45 (2734)
	AUC*	1.61 (4442)	0.78 (1677)		CCG	0.87 (1821)	0.60 (1139)
	AUA	0.37 (1025)	0.79 (1702)	Thr	ACU	0.96 (2294)	1.21 (2521)
Met	AUG	1.00 (4032)	1.00 (3118)		ACC*	1.49 (3545)	0.83 (1731)
Val	GUU	0.67 (1925)	1.40 (2912)		ACA	0.70 (1678)	1.25 (2607)
	GUC*	1.14 (3248)	0.79 (1635)		ACG	0.85 (2015)	0.71 (1486)
	GUA	0.37 (1061)	0.77 (1598)	Ala	GCU	1.00 (3271)	1.31 (3013)
	GUG*	1.82 (5203)	1.05 (2177)		GCC*	1.38 (4515)	0.80 (1840)
Tyr	UAU	0.50 (1151)	1.13 (1851)		GCA	0.71 (2315)	1.28 (2935)
	UAC*	1.50 (3424)	0.87 (1414)		GCG*	0.92 (3009)	0.62 (1417)
His	CAU	0.65 (1279)	1.23 (1936)	Cys	UGU	0.79 (1441)	1.08 (1673)
	CAC*	1.35 (2642)	0.77 (1216)		UGC*	1.21 (2188)	0.92 (1416)
Gln	CAA	0.71 (2117)	1.18 (3336)	Trp	UGG	1.00 (2015)	1.00 (1293)
	CAG*	1.29 (3842)	0.82 (2309)	Arg	CGU*	1.60 (2637)	0.93 (1267)
Asn	AAU	0.79 (2261)	1.28 (3935)		CGC*	1.88 (3105)	0.61 (830)
	AAC*	1.21 (3495)	0.72 (2199)		CGA	0.99 (1639)	1.03 (1414)
Lys	AAA	0.62 (2293)	1.10 (4690)		CGG	0.71 (1176)	0.63 (858)
	AAG*	1.38 (5114)	0.90 (3805)		AGA	0.34 (567)	1.62 (2220)
Asp	GAU	0.87 (3333)	1.24 (4539)		AGG	0.48 (795)	1.18 (1612)
	GAC*	1.13 (4308)	0.76 (2767)	Gly	GGU*	1.40 (3673)	1.18 (2195)
Glu	GAA	0.56 (2716)	1.13 (6131)		GGC*	1.46 (3819)	0.80 (1487)
	GAG*	1.44 (6946)	0.87 (4759)		GGA	0.66 (1742)	1.39 (2572)
					GGG	0.48 (1252)	0.62 (1158)

Comparison of codon usage frequencies between highly and lowly expressed sequences of *T. siginata* genes. AA: amino acid; N: number of codons; RSCU: Relative synonymous codon usage. Codon usage was compared using Chi squared contingency test to identify optimal codons. Asterisk denote codons that occurred significantly more often (*P* <0.01).

## Discussion

Codon usage bias is an important and complex evolutionary phenomenon, and it exists in a wide variety of organisms, from prokaryotes, to unicellular and multicellular eukaryotes. Some hypotheses are proposed to explain the origin of codon usage bias, among which neutral theory [38] and the selection-mutation-drift balance model [27,39] are the most representative ones. According to neutral theory, mutations at degenerate coding positions should be selectively neutral, thus resulting in random synonymous codon choice. In the selection-mutation-drift model, codon bias is thought to be determined by a balance between mutation pressure, genetic drift, and weak selection. In other words, if a gene experiences a highly selective pressure, such as high expression, it may be inclined to stronger codon usage bias. However, in recent years, with the completion of genome projects of many organisms, the two hypotheses are not sufficient to explain codon usage anymore. Many other factors have been reported to influence CUB, including gene length [11], GC-content [40,41], recombination rate [40,42,43], gene expression level [11,18,42], RNA structure [44–46], protein structure [47], intron length [48], population size [49], evolutionary age of the genes [50], environmental stress [51], the hydrophobicity and the aromaticity of the encoded proteins [52,53], and so on. In this study, the factors involved in shaping codon usage of the *Taenia saginata* genome at least includes gene expression level, gene compositional constraint, protein length, as well as the hydrophobicity of each protein (slightly).

Nucleotide composition could be one of the most important factors in shaping codon usage among genes and genomes. GC-rich organisms, such *as Bacteria, Archea, Fungi. Triticum Aestivum, Hordium vulgare and Oryza sativa* [36,54], tend to use G or C in the third position. And meanwhile, AT-rich organisms show a preference for A or T in third position, such as *Onchocerca volvulus, Mycoplasma capricolum* and *Plasmodium falciparum* [55–57]. The genomic G + C content for *T. saginata* is 43.61%. Although the genome would thus appear to be slightly A + T rich, overall codon usage is biased toward C- and G-ending codons (Table 2), this is similar to that in *Giardia lamblia* [13].

Previous studies have found significant negative correlations between protein length and CUB in variety of organsims, such as *yeast* [58], *Caenorhabditis elegans* [11], *Drosophila melanogaster* [41*]*, *Arabidopsis thaliana* [11] and *Silene latifolia* [59]. Similar results have also been found in *T. saginata*. There is an explanation proposed by Moriyama and Powell for this phenomenon: namely, if shorter proteins could perform similar functions to those of longer ones, longer proteins become energy-expensive and disadvantageous, thus the selection constraint acts to reduce the size of highly expressed genes, dominantly determines the relationship between codon bias and gene length [60].

As we know, it is difficult to quantify the expression level of genes in a differentiated multicellular eukaryote, where genes are expressed at different levels in different tissues and at different developmental stages. In the *T. saginata* genome, the expression level of an individual gene is lacking. It is known that EST counting is efficient for assessing gene expression level. Nevertheless, due to the limitation of EST numbers and rough prediction of gene expression level by counting ESTs, so we use the “Codon Adaptation Index” to evaluate the expression level of examined genes. CAI has been widely used to examine the expressivities of genes by many researchers and has now been considered as a well-accepted measure of gene expression [8,9].

In this study, we identified 23 codons as the optimal codons. Most of all optimal codons in the *T. saginata* genome end with G or C. This is very similar to the pattern observed in other eukaryotic genomes, such as *Dictyostelium discoideum* [61]*, D. melanogaster* [62]*, C. elegans* [18]*, Giardia lamblia* [13] *and Schizosaccharomyces pombe* [34]. The identification of optimal codons may provide useful clues for molecular genetic engineering and evolutionary studying.

## Conclusions

For the first time, we have reported the pattern of codon usage bias in the *T. saginata* genome and its causative factors. Evidence suggests that the codon usage pattern in *T. saginata* appears to be the result of a complex equilibrium between different forces, namely mutation bias, natural selection, the GC content of genes, protein length, gene expression level and hydropathicity. Meanwhile, 23 optimal codons were identified, all of which ended with either a G or C residue, this will be useful for cloning and expression of foreign genes in the organism. Such information from this study will provide a better understanding of the characteristics of synonymous codon usage in *T. saginata* and its molecular evolution, and provide a new resource to underpin the development of urgently needed treatments and control.
